# MAP4 Mechanism that Stabilizes Mitochondrial Permeability Transition in Hypoxia: Microtubule Enhancement and DYNLT1 Interaction with VDAC1

**DOI:** 10.1371/journal.pone.0028052

**Published:** 2011-12-02

**Authors:** Ya-dong Fang, Xue Xu, Yong-ming Dang, Yi-ming Zhang, Jia-ping Zhang, Jiong-yu Hu, Qiong Zhang, Xia Dai, Miao Teng, Dong-xia Zhang, Yue-sheng Huang

**Affiliations:** 1 State Key Laboratory for Trauma, Burn and Combined Injury, Institute of Burn Research, Southwest Hospital, Third Military Medical University, Chongqing, China; 2 The No. 324 Hospital of PLA, Chongqing, China; 3 Department of Plastic and Cosmetic Surgery, Xinqiao Hospital, Third Military Medical University, Chongqing, China; 4 Department of Plastic and Cosmetic Surgery, Southwest Hospital, Third Military Medical University, Chongqing, China; Thomas Jefferson University, United States of America

## Abstract

Mitochondrial membrane permeability has received considerable attention recently because of its key role in apoptosis and necrosis induced by physiological events such as hypoxia. The manner in which mitochondria interact with other molecules to regulate mitochondrial permeability and cell destiny remains elusive. Previously we verified that hypoxia-induced phosphorylation of microtubule-associated protein 4 (MAP4) could lead to microtubules (MTs) disruption. In this study, we established the hypoxic (1% O_2_) cell models of rat cardiomyocytes, H9c2 and HeLa cells to further test MAP4 function. We demonstrated that increase in the pool of MAP4 could promote the stabilization of MT networks by increasing the synthesis and polymerization of tubulin in hypoxia. Results showed MAP4 overexpression could enhance cell viability and ATP content under hypoxic conditions. Subsequently we employed a yeast two-hybrid system to tag a protein interacting with mitochondria, dynein light chain Tctex-type 1 (DYNLT1), by hVDAC1 bait. We confirmed that DYNLT1 had protein-protein interactions with voltage-dependent anion channel 1 (VDAC1) using co-immunoprecipitation; and immunofluorescence technique showed that DYNLT1 was closely associated with MTs and VDAC1. Furthermore, DYNLT1 interactions with MAP4 were explored using a knockdown technique. We thus propose two possible mechanisms triggered by MAP4: (1) stabilization of MT networks, (2) DYNLT1 modulation, which is connected with VDAC1, and inhibition of hypoxia-induced mitochondrial permeabilization.

## Introduction

Mitochondria are membrane enclosed organelles found in most eukaryotic cells [1]. The organelle consists of components or compartments that carry out specialized functions. These compartments or regions include the outer and inner membranes, intermembrane space, cristae, and matrix. Mitochondria are crucial for cellular function and have been described as “cellular powerhouses” because they generate most of the cell's ATP supply, which is used as the most important source of chemical energy. In addition to supplying cellular energy, mitochondria may play critical roles in a wide range of cytophysiological processes such as cell signaling, differentiation, cell death, as well as control of the cell cycle and growth [2]. Mitochondria have been implicated in several human diseases and cardiac dysfunction [3].

The induction of mitochondrial permeability transition (mPT) can cause mitochondrial depolarization to the point where the mitochondrial membrane potential (MMP) is abolished. When the MMP is lost there is an uninhibited flow of protons and various molecules across the outer mitochondrial membrane [4], [5]. The loss of the MMP also interferes with the production of ATP because the mitochondrion must have an electrochemical gradient to provide the driving force for ATP production. During the cell damage resulting from severe hypoxemic injury of the organism, such as a heart attack or severe burn, an induced permeabilization can severely reduce ATP production and even cause ATP synthase to start hydrolyzing ATP rather than producing it [6]. Many molecular chaperones are associated with mPT stabilization [7]. An increase in MMP is one of the key events in apoptotic and necrotic cell death that is purportedly regulated by various channels, for example the voltage-dependent anion-selective channel (VDAC), an outer membrane porin that also mediates the exchange of metabolites and energy between the cytosol and the mitochondria. Recent studies by Bernardi [8] and Molkentin [9] have raised doubts about the essential nature and involvement of some VDAC isoforms in the outer membrane mPT pore because knockout mice continue to express mPT pore activity. Nevertheless, VDAC continues to be put forward as a part of the permeabilization mechanism in normal cells; it also may be a component of the apoptotic machinery responsible for the release of cytochrome c, and thus an apoptosis-inducing factor [10], [11], [12].

Mitochondria are distributed with the aid of the cytoskeleton. Microtubules (MTs) are polymers of α- and β-tubulin dimers and have been assigned many functional roles in protein synthesis, intracellular trafficking, mitosis, cytokinesis, intracellular signaling, and cell fate determination [13], [14], when the ischemia-reperfusion and calcium overload occurring, the MTs are more prone to damage than the actin filaments [15]. Microtubule-associated proteins (MAPs) bind to tubulin subunits that make up MTs in order to regulate their stability. A variety of MAPs have been identified in different cell types and they perform various functions, for example, the fine tuning of MT dynamics to stabilize and destabilize MTs when guiding MTs towards specific cellular locations, MT cross-linking, and mediating interactions between MTs and other proteins [16], [17], [18]. MAP4 is found in nearly all cell types and is responsible for stabilization of MTs [19]. Takahashi et al. [20] reported that overexpression of MAP4 caused a shift of tubulin dimers to a polymerized fraction and formed dense, stable MT networks; overexpression also caused elevated tubulin expression and altered MT network properties [21]. Hypoxic stress can influence cell state whereby MAPs may be induced to act in a protective role by influencing MTs. Cortical neurons thrive under hypoxic conditions (1% O_2_) for significantly longer (7–14 days) than neurons cultured under ambient conditions (20% O_2_). One possible explanation is that this is due to the expression of MAP2 and the robust development of dendritic structure [22]. In contrast, our previous study [23] showed that hypoxia decreased cell viability and hypoxia-induced MAP4 phosphorylation lead to MT network disruption and an increase in free tubulin.

MTs function in concert with specialized dynein motors that are oriented such that the light chain portion is attached to cell organelles (e.g. mitochondria) and the dynamic portion is attached to MTs. Cytoplasmic dynein is the major motor protein complex responsible for MT-based motile processes. Dynein is an approximately twelve subunit complex consisting of two heavy chains, two intermediate chains anchored to its cargo, four smaller intermediate chains, and several light chains [24], [25], [26]. Schwarzer et al. [27] reported that Dynein light chain Tctex-type 1 (DYNLT1) slightly increases the voltage-dependence of VDAC1, indicating that DYNLT1 can modulate channel properties.

The above data indicate that under hypoxic conditions the disruption of MT networks may be a deciding factor in mitochondrial permeabilization and that MAP4 is involved as a potential modulator. We hypothesized that MAP4 may play a cytoprotective role by stabilizing MTs and by modulating DYNLT1, which is connected to VDAC1 and responsible for mPT induction and an MMP decrease. We show that MAP4 overexpression can alleviate the loss of ATP and DYNLT1 can diminish mPT by interacting with VDAC1 during hypoxia. Thus, we provide new insights into a MAP4 mechanism that stabilizes mitochondria and improves cell viability.

## Results

### Increase in the pool of MAP4 contributes to an increase in the structure of cytoplasmic MTs in both of cardiomyocytes and HeLa cells

**Figure 1:**
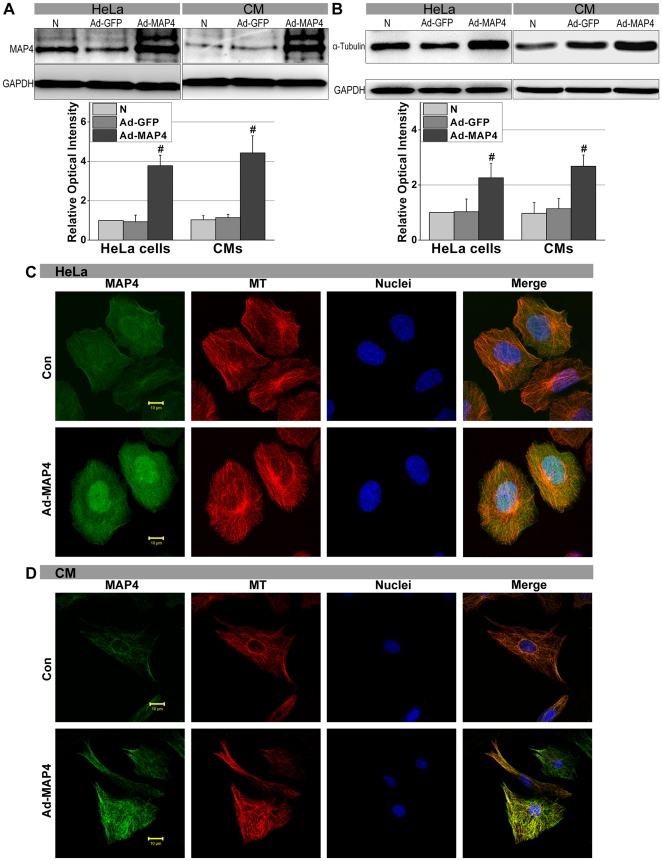
MAP4 overexpression increases the amount of α-tubulin and MT networks. **A** and **B**, Western blots showed that the expression of MAP4 and gross quantity of α-tubulin in HeLa cells and CMs after Ad-MAP4 was elevated after transfection. *Graphs* represent the mean±SEM of the relative optical density signals for three separate experiments (*n* = 3). N - non-transfected cells. # *P*<0.01 vs. N and Ad-GFP, Student's *t* test analysis. Cytosol GAPDH was chosen as the internal control. **C** and **D**, Immunofluorescent confocal micrographs of HeLa cells and CMs. Micrographs show that cells contain a larger amount of MAP4 (*FITC-green*) and more luxuriant MT network structure (*TRITC-red*) after Ad-MAP4 transfection compared with Con (Non-transfected cells). Scale bar, 10 µm.

In most cells, MAP4 can promote the addition of cytoplasmic tubulin dimers to the ends of MTs, consequently increasing the amount of MTs. We transfected neonatal rat ventricular cardiomyocytes (CMs) and HeLa cells with an adenovirus vector containing MAP4 cDNA (Ad-MAP4; +MAP4-CMs, +MAP4-HeLa cells). Western blot analysis indicated that the expression of dephosphorylated MAP4 was significantly elevated in +MAP4-CMs and +MAP4-HeLa cells, whereas no such differences were seen in non-transfected cells (N group) or cells transfected with Ad-GFP (Ad-GFP group) (Figure 1A, *P*>0.05). We chose α-tubulin as representative of the cytoplasmic tubulin pool. The constitutive quantity of α-tubulin in +MAP4 -CMs and -HeLa cells after transfection was much higher than that seen in the N and Ad-GFP groups (Figure 1B, *P*<0.01).

Confocal laser microscopy suggested that the amount of MAP4 (*FITC-green*) was much higher and the structure of MTs (*TRITC-red*) more luxuriant in +MAP4 (CMs and HeLa cells) than those in control cells (non-transfected). The merged images indicate that a plentiful amount of MAP4 was inserted into the MT structure, and apparently promoted the assembly of cytoplasmic MTs (Figure 1C, 1D).

### Elevated cytoplasmic MAP4 alleviates hypoxia-induced MTs disruption

**Figure 2:**
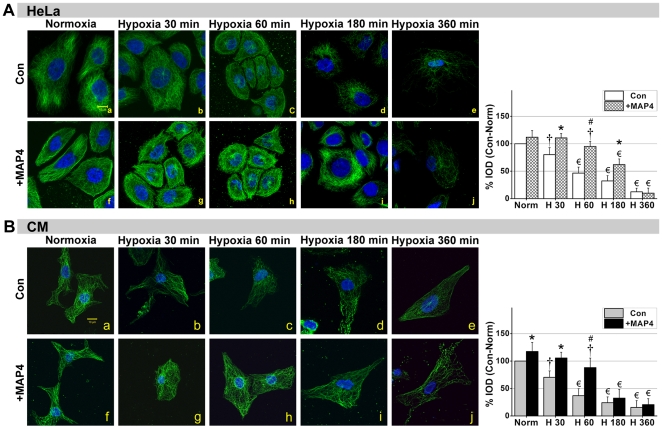
MAP4 overexpression alleviates MT disruption during the early stages of hypoxia. Immunofluorescent confocal micrographs of +MAP4 and Con (Non-transfected) HeLa cells (**A**) and CMs (**B**) under normoxia and hypoxia. Micrographs show the changes in MTs as hypoxia duration is increased. G*raphs* to the right represent the corresponding % Integral optical density of MTs (*green*) (IOD; values were normalized as percentage after comparison with normal, which were set to 100%; *n* = 3). Results show that the structure of MTs was significantly disrupted after 30 min of hypoxia in Con groups, while in +MAP4 groups this disruption was less apparent until 60 min. +MAP4 cells seemed to contain more MTs than Con cells undergoing the same level of hypoxia, especially at the earlier time points (≤60 min in CMs; ≤180 min in HeLa cells). All values are mean±SEM. * *P*<0.05, # *P*<0.01, vs. Con at each time point, Student's *t* test analysis; † *P*<0.05, € *P*<0.01, vs. Norm (Normoxia) within each group, One-way ANOVA followed by Tukey's post-hoc tests. Scale bar, 10 µm.

Tubular complexes are formed with α/β-tubulin heterodimers in the cytoplasm, and a dynamic balance exists between the assembly and disassembly of MTs. Hypoxia can destabilize this balance and instantly promote disassembly, even in the early stages of hypoxia (<30 min). The confocal images in Figure 2A and 2B show that after 30 min of hypoxia the structure of the MTs (*FITC-green*) was severely disrupted in both CMs and HeLa Con (non-transfected) groups, whereas in the +MAP4 groups, this collapse was retarded (Figure 2B-i), especially during the early stage of hypoxia. Figure 2B-c illustrates that the MT disruption was initiated around the periphery of the cell adjacent to the cytomembrane (60 min, *P*<0.01); this disruption then spread to entire cell as the hypoxic duration increased. Interestingly, in the early hypoxia condition MTs assembly in +MAP4 HeLa cells seemed enhanced near the cytomembrane (Figure 2A-g, h) but as hypoxia continued the MT network was more concentrated within the cytoplasm (Figure 2A-i) and subsequently faded away after 360 min of hypoxia (Figure 2A-j).

Quantification of fluorescent intensity levels showed that MT networks in +MAP4 CMs cells remained significantly higher versus control for at least 60 min of hypoxia (*P*<0.01), but that fluorescent intensity then dramatically declined and was not significantly different from control values at 180 min (*P*>0.05). Similar differences were noted in HeLa cells, although +MAP4 HeLa cells maintained significantly higher fluorescent values for longer (180 min) compared with control cells. Note that fluorescent intensity in +MAP4 normoxic cells were, in some cases, considerably higher than control values, particularly in the case of CMs.

### Protein-protein interactions between VDAC1 and DYNLT1; co-localization with MTs in the cytosol

**Figure 3:**
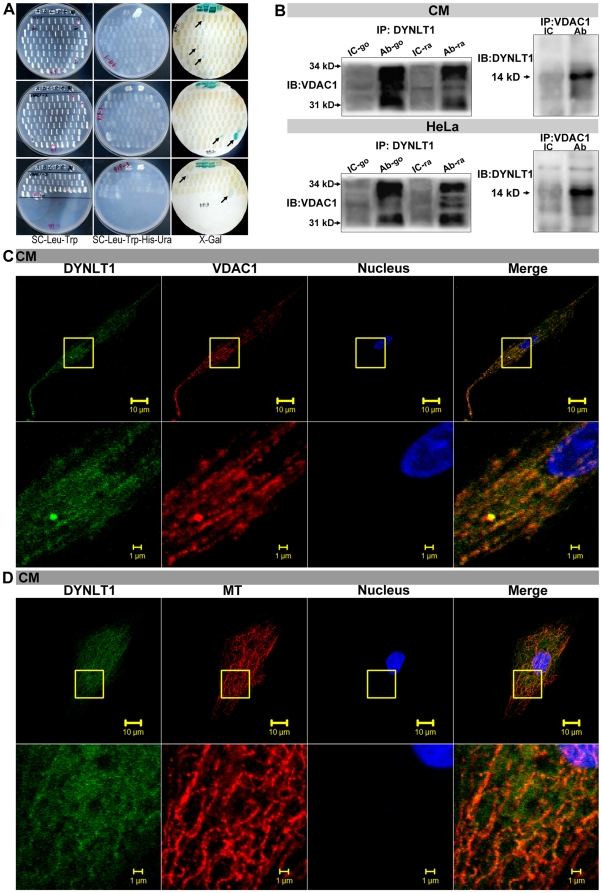
Interaction and co-localization of VDAC1, DYNLT1 and MTs. **A**, Example of *Yeast two-hybrid* technique. hVDAC1 was used as bait to tag new target molecules (arrows indicate the positive results detailed in Table 1). **B**, A co-IP approach followed by immunoblot analysis was used to test the protein-protein interaction between DYNLT1 and VDAC1. In whole-cell lysate of CMs and HeLa cells, abundant VDAC1 was detected in the antigen-antibody complex when applying anti-DYNLT1 (Ab-go / Ab-ra) compared with using IC-go / IC-ra. Anti-VDAC1 antibody co-immunoprecipitated with DYNLT1. IP- immunoprecipitate, IB- immunoblotting, Ab- antibody, IC- isotype control, go- goat, ra- rabbit. **C**, Immunofluorescent confocal micrographs showing the co-localization between DYNLT1 (FITC-green) and VDAC1 (TRITC-red) in CMs. **D**, Confocal micrographs showing the co-localization between DYNLT1 (FITC-green) and MTs (TRITC-red). Scale bar 10 µm. Areas in the boxed regions are shown at higher magnification, scale bar 1 µm.

In order to find a molecule that linked MT networks and mitochondria, a Yeast two-hybrid system was employed to tag potentially new molecules using hVDAC1 as bait. The final candidate tested was DYNLTD1 (Figure 3A, Table 1). DYNLT1 is an integral component of the dynein complex, via the dynein heavy chain, interacts with MT networks [24], [26], [28], [29]. Applied bioinformatics analysis, we speculated that there was a possible connection between DYNLT1 and VDAC1. As shown in Figure 3B, whole-cell CM and HeLa cell lysates contained a great deal of VDAC1 in an antigen-antibody complex based on a goat / rabbit anti-DYNLT1 antibody application. In a separate experiment using an anti-VDAC1 antibody, we showed that DYNLT1 was also detected in the co-immunoprecipitate.

**Table 1:** Tagging of interaction protein by yeast two-hybrid.

Bait Protein	Prey Protein	Prey Gene	NCBI_AC	Coding region	ORF	Report Gene	Coding site
VDAC1	DYNLT1	Homo sapiens dynein, light chain,1 (DYNLC1)	NM_006519	Yes	yes	LHU	1–559
VDAC1	APOB	Homo sapiens apolipoprotein B (including Ag(x) antigen) (APOB)	NM_000384	Yes	no	HU	13340–13923
VDAC1	PTPRH	Homo sapiens protein tyrosine phosphatase, receptor type, H (PTPR H), mRNA	NM_002842	Yes	yes	HU	2951–3539

Our next question concerned the morphological relationship between these three factors: DYNLT1, VDAC1 and MTs. Confocal images clearly showed that considerable overlap existed in the distribution of DYNLT1 (*FITC-green*) and VDAC1 (*TRITC-red*) (Figure 3C), and thus demonstrated their co-localization and the link between them. Imaging showed DYNLT1 (*FITC-green*) was widely distributed in the cytoplasm and the density of DYNLT1 was obviously higher along MTs (*TRITC-red*) (Figure 3D). Previous studies have proposed that DYNLT1 is involved in Dynein formation, whose main function is cargo transportation. Thus, our results indicate that DYNLT1, VDAC1 and MTs are co-localized within the cytoplasm.

### MAP4 overexpression leads to elevated expression of cytoplasmic DYNLT1

**Figure 4:**
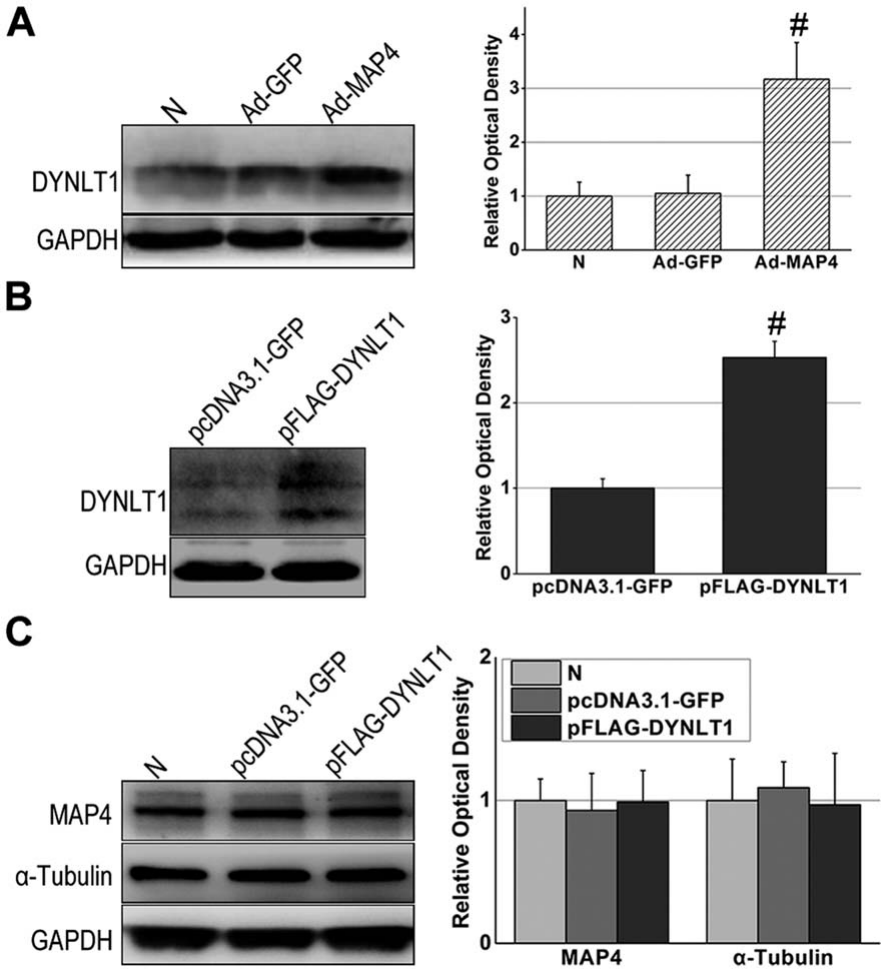
MAP4 overexpression leads to the elevated expression of DYNLT1. **A**, Immunoblot of DLNLT1 after MAP4 transfection. HeLa cells with MAP4 overexpression (Ad-MAP4) showed an elevated expression of DYNLT1 compared with non-transfected cells (N) and Ad-GFP transfected cells (Ad-GFP). # *P*<0.01 vs. N and Ad-GFP. **B**, Immunoblot of DYNLT1 after transient transfection of the plasmid. DYNLT1 was overexpressed in pFLAG-DYNLT1 cells. * *P*<0.05 vs. pcDNA3.1-GFP. **C**, Immunoblot of MAP4 and α-tubulin following up-regulation of DYNLT1 (pFLAG-DYNLT1). There seemed no influence on MAP4 and α-tubulin levels. *Graphs* represent the mean±SEM (*n* = 3) of the relative optical density signals.

The above data showed that overexpression of MAP4 led to elevated expression of tubulin (Figure 1B). We used western blot analysis to determine if +MAP4 also resulted in an increased expression of DYNLT1 (Figure 4A). A significantly elevated expression of DYNLT1 was also detected in +MAP4 HeLa cells (*P*<0.01). Transient transfection of a plasmid to increase DYNLT1 in HeLa cells (Figure 4B) shows that DYNLT1 was overexpressed accordingly, but there appeared no concomitant increase in MAP4 or α-tubulin (Figure 4C).

### MAP4 overexpression contributes to the maintenance of hypoxic energy metabolism

**Figure 5:**
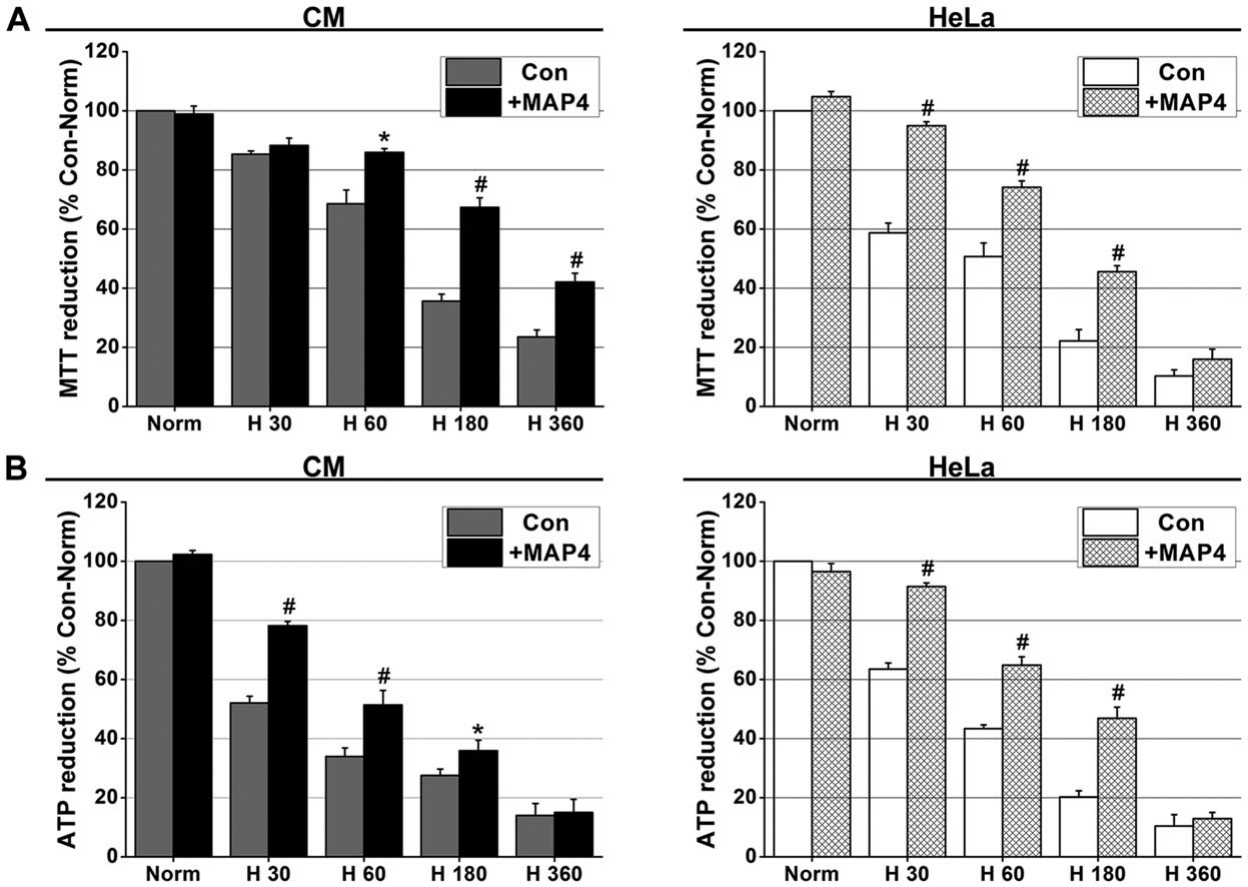
MAP4 overexpression contributes to cellular viability (measured by MTT) and energy metabolism maintenance (measured by ATP) during hypoxia. **A**, MTT reduction in +MAP4 groups (CMs and HeLa cells) was less when compared with Con (non-transfected) cells. **B**, ATP reduction in +MAP4 groups was also less when compared with Con cells. Values were compared to normal values (Norm; first bar), which were set to 100% and the other values normalized accordingly. *Graph* represents the mean±SEM (*n* = 6, Separate six experiments) of the relative luminescence signals. * *P*<0.05, # *P*<0.01 vs. Con.

Con and +MAP4 groups of CMs and HeLa cells were established and exposed to hypoxic conditions for 30, 60, 180 and 360 min. The relative cellular viability of the cultured cells was tested using the MTT method. Figure 5A illustrates that the +MAP4 CMs were more viable than control CMs at all times after 60 min. +MAP4 HeLa cells showed an earlier resistance to hypoxia compared to controls and CMs in MTT reduction after 30 min of hypoxia (*P*<0.01), but differences were not statistically significant at 360 min (*P*>0.05). Figure 5B illustrates that in either cell category, the relative ATP content in the +MAP4 group was significantly higher than that in the control cells between 30 and 180 min, after which ATP quantity fell to low levels and by 360 min no significant difference was detected.

There is a degree of variability between CMs and HeLa cell responses at two time points: 30 and 360 min in Figure 5A. This might be due to inherent differences between the HeLa and CM cells in their ability to withstand hypoxia.

### DYNLT1 knockdown aggravates hypoxia-induced mitochondrial damage: permeabilization, MMP collapse and metabolic viability reduction

**Figure 6:**
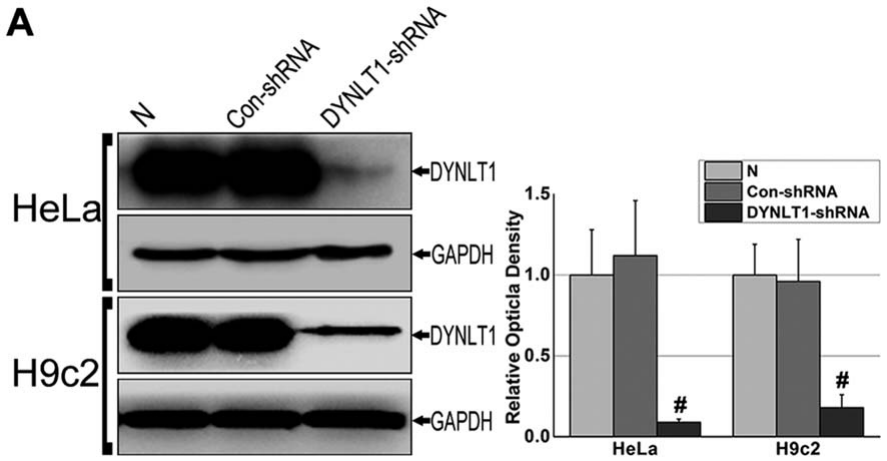
Verification of DYNLT1 knockdown in HeLa and H9c2 cell lines. H9c2 is a subclone of the original clonal cell line derived from embryonic BD1X rat heart tissue; we chose H9c2 and HeLa cell lines to establish stable cell lines with low expression of DYNLT1 (−DYNLT1) by RNAi technique. **A**, Immunoblot and quantified relative optical density analysis showed a stable low expression of DYNLT1 in cell clones using a RNAi approach (DYNLT1-shRNA) compared with non-transfected cells (N) and control plasmid DNA transfected cells (Con-shRNA). *Graph* represents the mean±SEM (*n* = 3). # *P*<0.01 vs. N and Con-shRNA.

**Figure 7:**
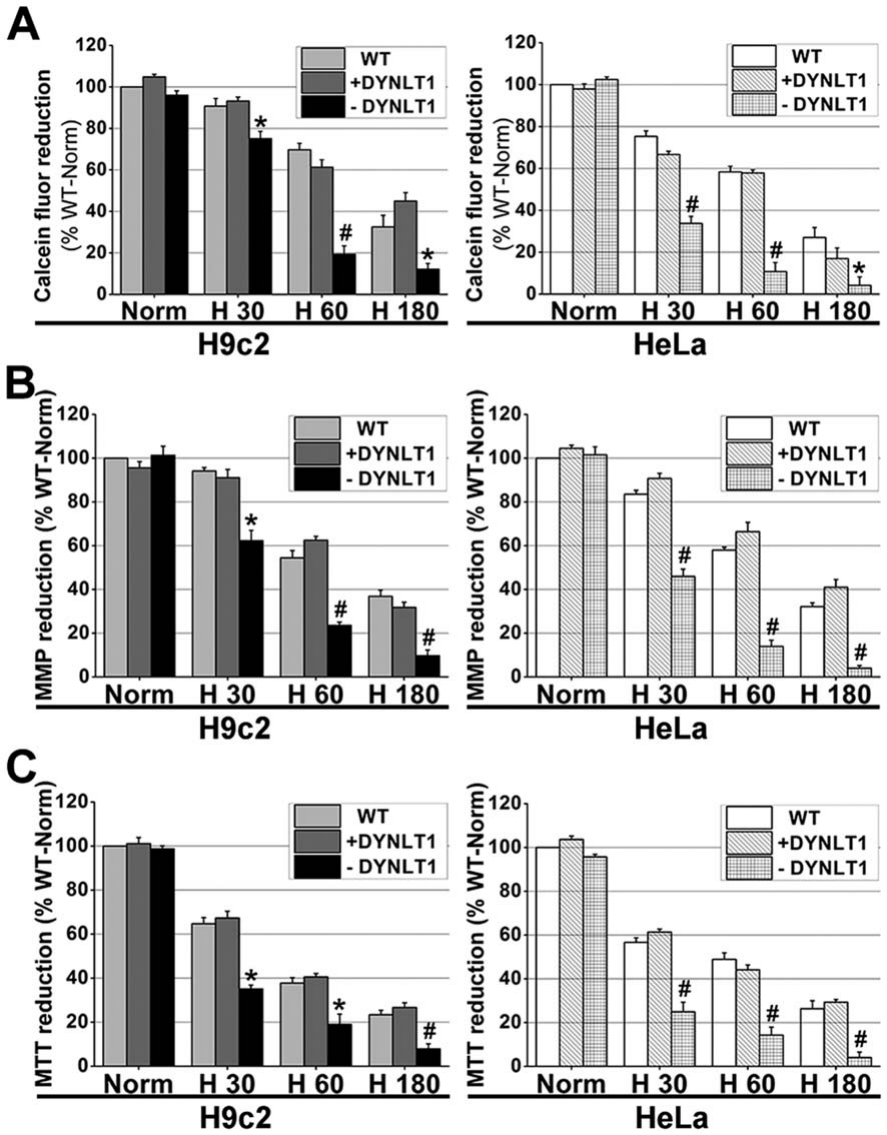
DYNLT1 knockdown aggravates hypoxia-induced damage of mitochondria: mPT induction, MMP disruption and reduction of cellular viability. **A**, −DYNLT1 (RNAi by DYNLT1-shRNA) groups showed a sharp reduction in calcein fluorescence after 60 min of hypoxia (cf. H30, H60 and H180); while +DYNLT1 (pFLAG-DYNLT1 transfected) cells showed no significant difference from WT (non-transfected) cells. **B**, −DYNLT1 cells showed a significant hypoxia-induced MMP damage, whereas +DYNLT1 cells appeared showed a slower decline in MMP that was similar to WT cells. **C**, −DYNLT1 cells showed a dramatic decrease in cell viability compared with WT and +DYNLT1 cells; WT and +DYNLT1 cells were not significantly different. Values were compared to Norm (first bar), which was set at 100% and the other values were normalized accordingly. *Graphs* represent the mean±SEM (*n* = 6). * *P*<0.05, # *P*<0.01 vs. WT group or +DYNLT1 group.

The H9c2 cell line, a subclone of the original clonal cell line derived from embryonic BD1X rat heart tissue, was chosen instead of CMs in order to establish stable cell lines with a knockdown of DYNLT1 (−DYNLT1) after the RNA interference technique. We used a shRNA vector to knockdown the DYNLT1 because of the confirmed protein-protein interactions between DYNLT1 and VDAC1, and then established and verified which subclones had a low and stable expression of DYNLT1 (i.e. HeLa-dD and H9c2-dD cells). The effect of DYNLT1 down-regulation was analyzed with Western blots (Figure 6). As shown in Figure 7A and 7B, the DYNLT1 knockdown aggravated hypoxia-induced MMP damage and mPT followed by mitochondrial injury; this damage was not ameliorated by +DYNLT1 (DYNLT1 overexpression). Figure 7C shows that the depletion of DYNLT1 (−DYNLT1) under hypoxic conditions caused a sharp decrease in relative cell viability. On the other hand, DYNLT1 overexpression did not effectively protect the cell from hypoxia-induced damage compared with the WT (non-transfected) group.

## Discussion

Hypoxic-ischemic injury is the result of oxygen lack, a condition that extensively occurs as a primary complication of heart dysfunction, stroke, and severe burn wounds. Mitochondria are crucial organelles that trigger or facilitate cell disruption under hypoxic conditions. Increasing attention has been directed towards mPT because induced permeabilization will cause matrix swelling, MMP loss and cellular energy dysmetabolism. VDAC has been proposed as an important channel located in the outer membrane. Among the three isoforms, VDAC1 is the most abundant porin isoform in mammals. Despite the controversy about VDAC1's essential involvement in mPT pore components, it is widely accepted that VDAC1 serves as master regulator of mPT and consequently mitochondrial function by allowing exchange of ions and metabolites between the intermembrane space and cytosol, and the release of apoptotic proteins, such as cytochrome C, into the cytosol. Therefore, an increasing number of recent studies have focused on VDAC as a means of protecting the organism against hypoxic damage [10], [11], [12].

MTs are an important element of the cytoskeleton that supports the distribution of mitochondria in the cytosol. Our previous study on CMs and HeLa cells [23] suggested that the collapse of MT networks develops quickly during hypoxia, such that, within 15 min after the onset of hypoxia the MT networks have begun partial depolymerization. This damage preceded cellular energy dysfunction. Nevertheless, the manner in which MTs function during hypoxia and the link between MTs and mitochondria remained elusive. We also observed that hypoxia-induced MAP4 phosphorylation could lead to MT network disruption and an increase in free tubulin [23]. This information suggested to us that MAP4 might be a protein potentially involved in regulating mitochondrial function through the MT pathway. Here we performed experiments to further determine the effect of MAP4 on MTs and showed that total cytoplasmic tubulin was up-regulated, and MT networks are enhanced in cells overexpressing MAP4 (Figure 1). These results are in agreement with previous reports by Sato et al. and Cheng et al. using adult cat CMs in vitro [21], [30]. Furthermore, we found dephosphorylated MAP4 overexpression could prevent MT disruption in hypoxia (Figure 2). These observations suggest that transient overexpression of MAP4 can be a protective factor to MTs. In addition, the up-regulated MT production and observed MT stabilization was associated with a relative maintenance of cellular energy metabolism during the early stages (<180 min) of hypoxia (Figure 5). These results suggest that inhibition of VDAC by tubulin binding might modulate MMP and restrict outer membrane permeability for ADP and ATP [31], [32]. Our research seems to be consistent with this when the interaction between MTs, VDAC1 and DYNLT1 are considered (see below).

MAP4 can modulate MTs to maintain mitochondrial function, and VDAC acts as a crucial protein during this procedure. Using Y2H technique (Figure 3A), we searched for a intermediate molecule linking mitochondria (VDAC) and MTs, and came up with DYNLT1 as a promising candidate (Figure 3B and 3C, Table 1). Dynein light chain Tctex-type 1 (DYNLT1) assists the intermediate chain, another component of dynein complex, to fulfill cargo binding function [24], [25], and plays a key role in multiple steps of hippocampal neuron development, including initial neurite sprouting, axon specification, and dendritic elaboration [33], [34]. DYNLT1 acts in an independent cargo adaptor role for dynein motor transport apart from other neuritogenic effects elicited by itself [35]. Although numerous reports have addressed dynein subunits, the mechanism of how they function with other molecules in the cytosol remains unclear. Schwarzer [27] reported on the protein-protein interactions between DYNLT1 and VDAC1 and this was supported by our immunofluorescence co-localization and immunoprecipitation experiments (Figure 3B and 3C), accordingly, we speculate that DYNLT1 may be one of the regulators of VDAC1. Based on the above data, we presume that DYNLT1 is a potential intermediate molecule, which can damage mitochondria via VDAC1 during the course of MTs disruption when hypoxia. This hypothesis was further strengthened by finding that there was a close association between DYNLT1, VDAC1 and MTs in the cytosol (Figure 3C and 3D).

As shown in Figure 1B, MAP4 overexpression can constitutively up-regulate tubulin, and, intriguingly, also heightens DYNLT1 expression in CMs and HeLa cells (Figure 4A). Our results posed two additional questions: 1. Will overexpression or inhibition of DYNLT1 effect mPT and energy metabolism during hypoxia? 2. Is the beneficial potency of MAP4 overexpression on energy metabolism due to the effect of MAP4 on DYNLT1? The western blots indicated that although elevated expression of MAP4 led to up-regulated expression of DYNLT1 and tubulin, DYNLT1 overexpression per se had no influence on tubulin and MAP4 levels (Figure 4C). However, DYNLT1-knockdown experiments showed a dramatic increase in sensitivity to hypoxia with a concomitant reduction in cell viability and MMP and mPT damage (Figure 7). These findings suggest a previously unknown mitochondrial mechanism of DYNLT1 regulation, possibly governed by MAP4. Hypoxic damage will be aggravated with the absence of DYNLT1, while its overexpression seems to have no effect. Given the fact that DYNLT1 is associated with MTs and interacts with VDAC, DYNLT1 regulation can be an independent way for MAP4 to effect mitochondrial stabilization.

**Figure 8:**
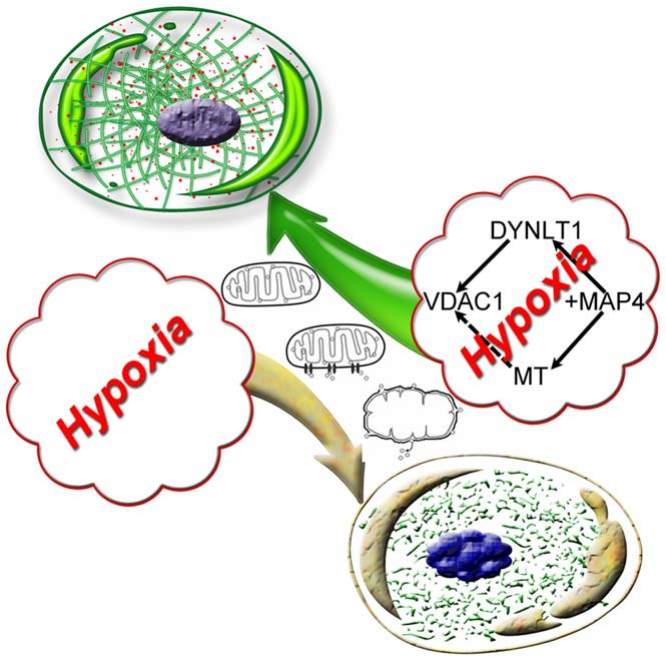
Model of MAP4, MTs and DYNLT1 interactions that might prevent hypoxia-induced cell damage. The proposed model was built to describe a different cell destiny with the absence or presence of a hypothetical modulation during hypoxia. MAP4 overexpression may be a trigger in stabilizing mitochondrial function by enhancing the structure of MTs and promoting DYNLT1 expression. We demonstrated that DYNLTI interacts with VDAC1, which is considered responsible for mPT and consequent cell death. MT enhancement might be another potential mediator by binding tubulin to VDAC1 in addition to its supporting role with mitochondria.

Our study proposes MAP4 mechanism for stabilizing mitochondrial function in hypoxia (Figure 8): (1) MAP4 stabilizes MT networks. (2) Overexpression MAP4 up-regulates DYNLT1, which interacts with VDAC1, and thus hypoxia-induced mPT is effectively inhibited. Although MAP4 overexpression only temporarily relieves energy dysmetabolism and enhances the cell's tolerance to hypoxia, these coordinated effects could be vital for cellular survival in cases of temporary severe hypoxia or hypoxia-reoxygenation, and thus may contribute to functional organ recovery in severe hypoxic-ischemic injury. However, there remains much work to be done exploring the precise mechanisms of MAP4 regulation, the interaction between tubulin and VDAC1, and especially, a new role of DYNLT1 in cytoprotection based on mitochondrial function.

## Materials and Methods

### Cell culture

All animal procedures were approved by the Institutional Animal Care and Use Committee of the Third Military Medical University (Permit Number SYXK-CQ-20070002) and adhered to the Principles of Laboratory Animal Care (NIH publication). Neonatal rat CMs were prepared according to the methods of Lange et al. [36]. CMs from 1- to 3-day-old Sprague–Dawley rats were plated at 5×10^6^ cells/60-mm dish and maintained for 48 h in DMEM/F12 with 5-bromodeoxyuridine (BrdU, 31 mg/L; Sigma), 10% (v/v) heat-inactivated fetal bovine serum (FBS), penicillin G (100 U/ml), and streptomycin (100 mg/ml) before hypoxia treatment. HeLa and H9c2 cell lines were obtained from the American Type Culture Collection (ATCC Manassas, VA, USA). HeLa cells were cultured in RPMI-1640 and H9c2 cultured in DMEM under standard mammalian cell conditions.

### Hypoxia treatment

Hypoxia was induced according to previous methods [37]. Briefly, hypoxic conditions were achieved by using an anaerobic jar (Mitsubishi, Tokyo, Japan) and vacuum glove box (Chunlong, Lianyungang, China). Serum-free medium was placed in a vacuum glove box filled with a gas mixture of 94% N_2_, 5% CO_2_, and 1% O_2_ (v/v) overnight and allowed to equilibrate with the hypoxic atmosphere. Cells were subjected to hypoxic conditions by replacing the normoxic medium with the hypoxic medium and the culture dishes then placed in the anaerobic jar.

### MAP4 recombinant adenovirus construction and transfection

To induce MAP4 overexpression, we constructed a recombinant adenovirus that expressed rat MAP4. The recombinant adenoviruses were prepared using the Adeno-XTM system (BD ClonTech, USA) according to the instructions. The transgene expression in CMs and HeLa cells was tested by western blots. CMs were maintained in DMEM/F12 and 10% FBS. HeLa cells were maintained in RPMI-1640 and 5% FBS. The medium was changed to medium without FBS, and cells were infected with adenoviruses at a multiplicity of infection of 100–200 particles/cell for about 36 h. The cells were then cultured in DMEM/F12 or RPMI-1640 with FBS before morphological or biochemical analysis.

### Immunofluorescence microscopy

Immunocytochemical staining was performed as described previously [38], [39]. Cells were cultured on coverslips (10-mm diameter) and stained, and then five fields chosen on each coverslip (2 across the top and bottom and 1 in the middle). After each treatment, cells were rinsed twice in prewarmed (37°C) PBS and then fixed in cold (−20°C) methanol for 3 min and soaked three times in cold acetone. Cells were rehydrated with PBS, blocked for 20 min with PBS containing 5% FCS and 0.1% bovine serum albumin (BSA; Sigma), and then incubated for 60 min with a mouse primary antibody. For immunofluorescence microscopy, antibodies were directed against MAP4 (1∶500; BD Biosciences), α-tubulin (1∶1000; Santa Cruz Biotechnology, Santa Cruz, CA), DYNLT1 (1∶500; Santa Cruz), VDAC1 (1∶500; Santa Cruz Biotechnology). Secondary antibodies used were FITC (fluorescein isothiocyanate) - and TRITC (tetramethylrhodamine isothiocyanate)-conjugated antibodies (Santa Cruz). Finally, counterstaining of nuclei was performed with 4,6-diamidino-2-phenylindole (DAPI; Biotium, Hayward, CA). The cells were observed and photographed with LSM 510 META laser confocal scanning microscope (Carl Zeiss, Germany). The fluorescence intensity of individual cells was measured and analyzed with Image-Pro Plus 6.0 (Media Cybernetics, Inc. USA). We randomly chose one intact cell per field and measured five cells per coverslip. Four coverslips (20 cells) from each time point for each group (Figure 2A and 2B) were analyzed by immunofluorescence and the whole experiment repeatedly three times (*n* = 3).

### DYNLT1 knockdown and establishment of stable cell clones

To reduce DYNLT1 expression [40], HeLa and H9c2 cells were seeded in 6-well plates in normal growth medium. Cells were grown to 50–70% confluency in antibiotic-free normal growth medium supplemented with FBS. A shRNA Plasmid DNA (shRNA strand constructs against hDYNLT1: A) 5′ - CUUCGGACUGUCUAUUUGA -3′, B) 5′ -GAAGAAUGGAGCUGGAUUA-3′ and C) 5′ - CCACAAAUGUAGUAGAACA -3′; sc-43319-SH, Santa Cruz, USA) solution was added directly to the dilute shRNA Plasmid Transfection Reagent (sc-108061, Santa Cruz). Cells were washed twice with shRNA Transfection Medium (sc-108062, Santa Cruz), and then 200 µl of shRNA Plasmid DNA/shRNA Plasmid Transfection Reagent Complex added drop-wise in order to cover the entire layer. Cells were incubated for 5–7 h at 37°C in a CO_2_ incubator or under conditions normally used to culture the cells. Following incubation, 1 ml of normal growth medium containing 2 times the normal serum and antibiotics (2× normal growth medium) was added to the medium and the cells incubated for an additional 18–24 hours under conditions normally used to culture the cells. The control shRNA Plasmids (sc-108060, Santa Cruz) encode a scrambled shRNA sequence that will not lead to the specific degradation of any known cellular mRNA.

We used puromycin [41] to select stable transfected cells, as follows: 48 hours post-transfection, the medium was aspirated and replaced with fresh medium containing puromycin at the appropriate concentration (2–4 µg/ml). Every 2–3 days the media was aspirated and replaced with freshly prepared selective media. The depletion levels of DYNLT1 were confirmed by Western blotting. We named the stable cell clones that underwent DYNLT1 knock-down as HeLa-dD and H9c2-dD.

### Construction of plasmids and transfection

Full-length human DYNLT1 was subcloned into pFLAG (pCMV-Tag 2C, Stratagene). Cell lines and control cell lines were developed by electroporating cells with 20 mg of pFLAG-DYNLT1 and pcDNA-3.1-GFP (control plasmid, ClonTech) respectively with a single pulse of 1 ms at 200 V. After 2 days incubation, the overexpression levels of DYNLT1 were examined by Western blotting.

### Yeast two-hybrid screen

The Hybrid Hunter™ two-hybrid system Kit (Invitrogen) was used. The yeast strain MaV203 was transformed with pDBleu-hVDAC1, pPC86-cDNA library and subsequently with a cDNA library (ProQuest™, Invitrogen) derived from human hepatocytes. Yeast cells were plated on selection plates lacking His and Trp. Primary positive colonies were replaced and tested for LacZ expression by filter assay. Prey plasmids of positive colonies were recovered, transformed into E. coli, and sequenced. To reproduce positive interactions in yeast, prey plasmids were re-transformed in MaV203 together with pDBleu-hVDAC1 or with control bait plasmids.

### Western blot analysis

Whole cell lysate were prepared [42] and analyzed by western blotting using primary antibodies to MAP4 (anti-MAP4 1∶ 500, Bethyl), α-tubulin (anti-α-tubulin 1∶1000, Santa Cruz), DYNLT1 (anti-DYNLT1 1∶500, Santa Cruz) and VDAC1 (anti-VDAC 1∶500, Santa Cruz). As a loading control, GAPDH was probed and visualized. Immunocomplexes were visualized and quantified with an enhanced chemiluminescence detection kit (Amersham Pharmacia, Piscataway, NJ), using horseradish peroxidase-conjugated secondary antibodies (1∶2000; Santa Cruz).

### Immunoprecipitation

To show the VDAC1 and DYNLT1 complex formation, CMs and HeLa cells (60 mm dish) were lysed in 300 µl RIPA (Sigma) buffer with 2 mM PMSF and a protease inhibitor cocktail. Anti-DYNLT1 (E-16, goat polyclonal Ab; H-60, rabbit polyclonal Ab, Santa Cruz) or anti-VDAC1 (FL-283 rabbit polyclonal Ab, Santa Cruz) antibodies were incubated with 150 µl cell lysate for 6 h at 4°C. Afterward the complexes were precipitated with protein A/G-Sepharose (Santa Cruz) overnight at 4°C. The precipitates were washed 3 times with PBS at 0°C, then probed with anti-VDAC1 or anti-DYNLT1 antibodies by western blotting.

### Quantification of ATP

CellTiter-Glo™ Luminescent Cell Viability Assay kits were used for ATP assay following the manufacturer's instructions. Briefly, treated cells were lysed in a 200 ml volume (4 mM EDTA, 0.2% Triton X-100) for 5 min; the assay buffer and substrate were equilibrated to room temperature. In 96-well plates, 100 ml of the assay reagent (25 mM HEPES pH 7.25, 300 mM Dluciferin, 5 mg/ml firefly luciferase, 75 mM DTT, 6.25 mM MgCl2, 625 mM EDTA and 1 mg/ml BSA) was added to each well and the contents mixed for 2 min on an orbital shaker to induce cell lysis. After 10 min incubation at room temperature, the luminescence was read on a Microplate Reader. ATP levels were standardized to cell number for protein levels using BCA assay.

### Cell viability assay

Relative cell viability was determined using MTT (3-(4, 5-dimethylthiazolyl-2)-2, 5-diphenyltetrazolium bromide; 5 mg/ml in PBS) [43], which was added at a final concentration of 125 mg/ml after treatment. The cells were incubated with MTT for 3 h at 37°C, solubilized in dimethyl formamide (50%; v/v) and SDS (20%; w/v), prior to absorbance measurements at 570 nm.

### Determination of Mitochondrial Membrane Potential

Mitochondrial membrane potential (MMP) was assessed using tetramethyl rhodamine methyl ester (TMRE, Invitrogen), a lipophilic cationic fluorescent probe that becomes localized within the mitochondria as a function of membrane potential [44], [45]. Cells grown on coverslips were loaded with 500 nM TMRE for 30 min at 37°C in Hank's balanced salt solution. Real time imaging of live cells was performed with a fluorescence imaging system (Leica DM6000 B, Leica, Germany). Dye loaded cells were maintained in a perfusion chamber (bath volume = 0.5 ml) mounted on the microscope stage. For detection of fluorescence, 568 nm excitation and 585 nm emission filter settings were used. Images were collected with an exposure time of 100 msec. Regions of interest (ROI) were selected within several cells in each experiment for measuring changes in MMP.

### Mitochondrial permeability transition

The mPT was measured by calcein-AM / CoCl_2_ staining as described previously by Petronilli et al [46], [47]. Briefly, cells were loaded with 1 µM calcein-AM ester and 1 mM CoCl_2_ in Hanks' solution containing 10 mM Hepes buffer (pH 7.4) for 0.5 h at 37°C. Cells were washed to remove the free calcein and Co^2+^, and then analyzed using flow cytometry (Epics XL-MCL, BECKMAN, USA) with an excitation at 490 nm and emission at 520 nm.

### Statistical analysis

Data are expressed as mean±SEM. SPSS 13.0 was used for statistical analysis and significance evaluated by one-way ANOVA followed by post-hoc tests and paired-samples T test. *P* values<0.05 were considered statistically significant.
